# Data on high performance supercapacitors based on mesoporous activated carbon materials with ultrahigh mesopore volume and effective specific surface area

**DOI:** 10.1016/j.dib.2018.04.057

**Published:** 2018-04-23

**Authors:** Yanhong Lu, Suling Zhang, Jiameng Yin, Congcong Bai, Junhao Zhang, Yingxue Li, Yang Yang, Zhen Ge, Miao Zhang, Lei Wei, Maixia Ma, Yanfeng Ma, Yongsheng Chen

**Affiliations:** a School of Chemistry & Material Science, Langfang Normal University, Langfang 065000, China; b The Centre of Nanoscale Science and Technology and Key Laboratory of Functional Polymer Materials, State Key Laboratory and Institute of Elemento-Organic Chemistry, College of Chemistry, Nankai University, Tianjin 300071, China

## Abstract

The data presented in this data article are related to the research article entitled “Mesoporous activated carbon materials with ultrahigh mesopore volume and effective specific surface area for high performance supercapacitors” (Lu et al., 2017) [1]. The detailed structure data of the prepared mesoporous activated carbon materials with ultrahigh mesopore volume and effective specific surface area and the electrochemical performance data of the corresponding supercapacitors are described.

**Specifications Table**

**Table:** 

Subject area	Chemistry
More specific subject area	Materials science: activated carbons (ACs)
Type of data	Table, image, text file, graph, figure and video
How data was acquired	X-ray photoelectron spectroscopy (XPS) spectrometer (AXIS HIS 165, Kratos Analytical); Nitrogen isotherm of N_2_ at 77 K (Micromeritics ASAP 2020); CHI660C electrochemical analyzer (Shanghai Chenhua Instruments Co., Ltd.); Battery test system (LAND CT2001A model, Wuhan LAND Electronics. Ltd.); P4000 electrochemical workstation (Princeton, USA)
Data format	Raw and analyzed
Experimental factors	Samples, assembled coin-type supercapacitor and calculation
Experimental features	Characterization of electrode material, assembly of device and measurement of electrochemical performance
Data source location	Langfang Normal University and Nankai University, China
Data accessibility	Data are presented in this article

**Value of the data**

• Detailed experimental data and procedures might be used for further scientific development and further studies.
• Surface element composition data and pore size distribution curves could be used for more scientific analysis of the microstructure of carbon materials, and helpful for material design and optimization.
• The calculation method for effective specific surface area and theoretical capacitance could be used to evaluate reliably the material's performance used for double layer electrochemical capacitor without fabricating real and industry standard devices.
• Electrochemical performance data of other materials including the reference material than the optimal material can be used to compare together and used for the optimization of material design.

## 1 Data

**Fig. 1:**
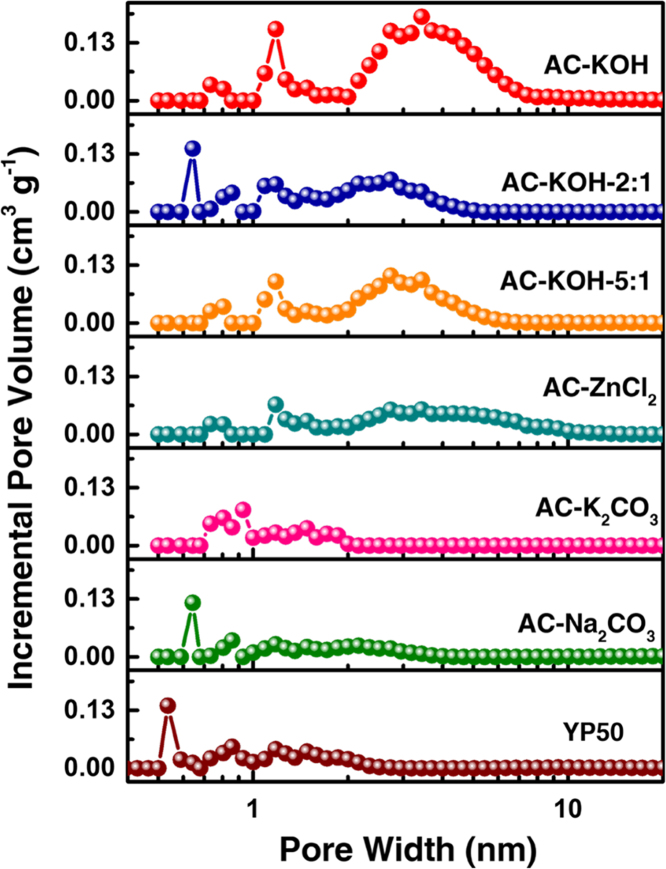
Pore size distribution of a series of prepared ACs with different activation agents/ratios and the compared commercial YP50 based on the NL-DFT method from the nitrogen adsorption data.

**Fig. 2:**
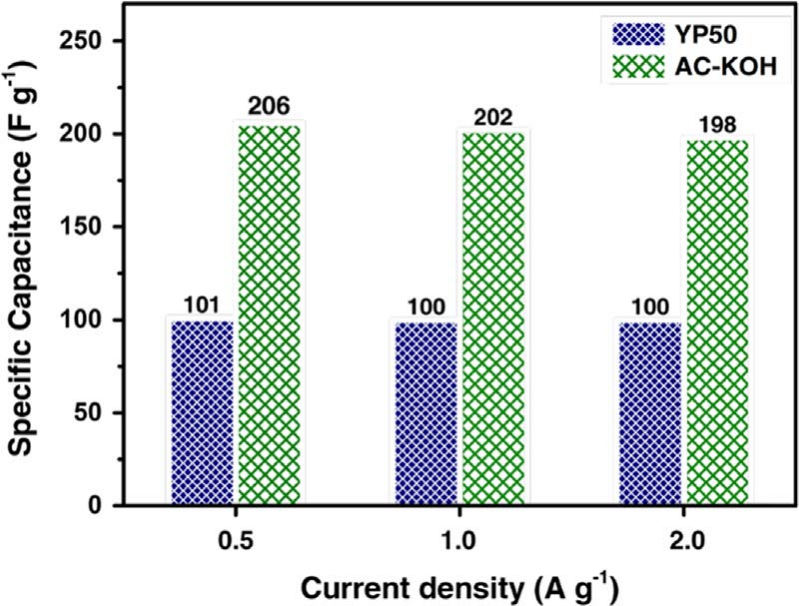
Capacitance performance of the supercapacitor with prepared AC-KOH and commercial YP50 in TEABF_4_/AN electrolyte system tested at current densities from 0.5 to 2 A g^-1^.

**Fig. 3:**
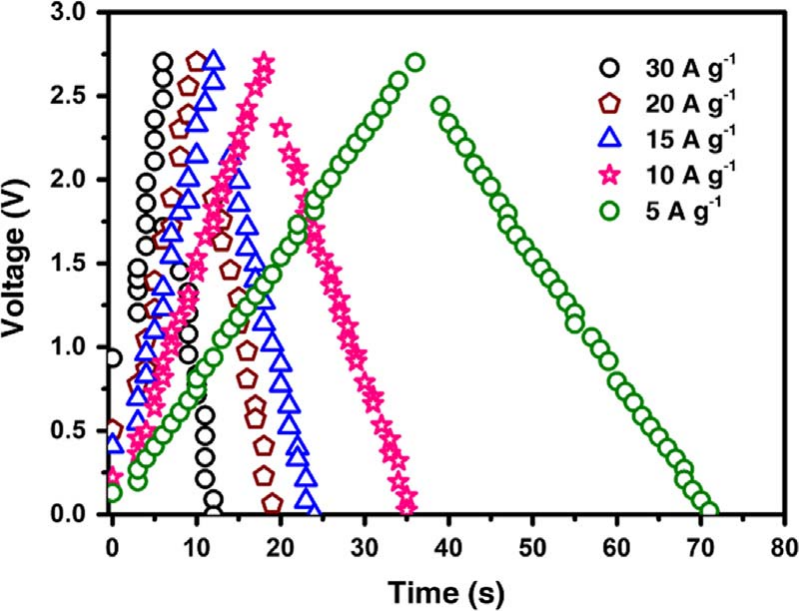
Galvanostatic charge/discharge curves for AC-KOH based supercapacitor tested at current density of 5, 10, 15, 20 and 30 A g^-1^, respectively.

**Fig. 4:**
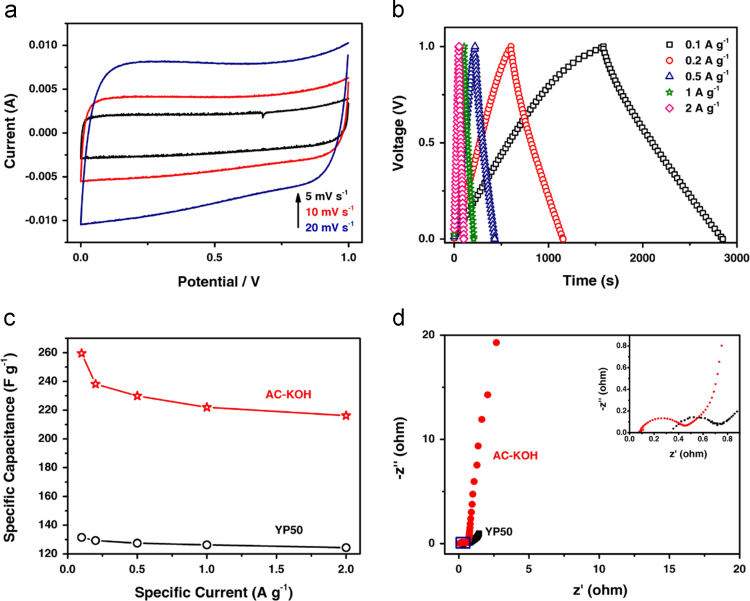
Electrochemical performance of AC-KOH and YP50 based supercapacitors in 6 M KOH electrolyte system. (a) CV curves of AC-KOH based supercapacitor measured at the scan rates of 5, 10 and 20 mV s^-1^ in the potential range of 0−1.0 V. (b) Galvanostatic charge/discharge curves for AC-KOH based supercapacitor tested at current densities from 0.1 to 2 A g^-1^. (c) Rate performances and (d) Nyquist plots of AC-KOH and YP50 based supercapacitors. The inset in (d) is the enlarged one of the rectangular region.

**Fig. 5:**
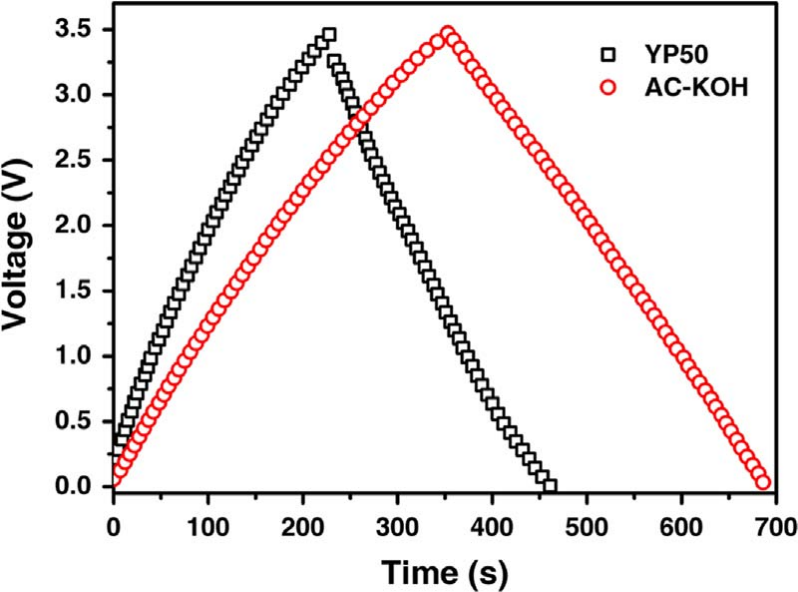
Galvanostatic charge/discharge curves for AC-KOH and YP50 based supercapacitors in EMIMBF_4_ electrolyte system tested at current density of 1 A g^-1^.

**Fig. 6:**
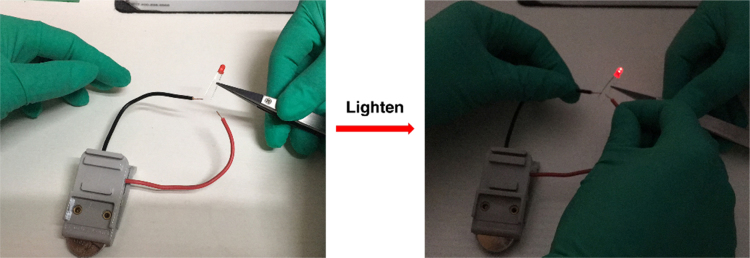
The optical images of a red LED before and after lightened by a charged supercapacitor with AC-KOH as the electrode material and EMIMBF_4_ as the electrolyte.

In this data article, the detailed structure and electrochemical data of the prepared mesoporous activated carbon materials (AC-KOH) and the control samples are presented. The data include the surface element composition (Table 1), pore size distribution (PSD) curves (Fig. 1), the calculation method of the effective specific surface (E-SSA) (Table 2), the electrochemical performance data in organic (TEABF_4_/AN), aqueous (KOH) and ion liquid (EMIMBF_4_) electrolyte systems (Fig. 2, Fig. 3, Fig. 4, Fig. 5). The photograph and video for lighting a LED using the AC-KOH based supercapacitor are demonstrated (Fig. 6). In addition, the structure and electrochemical data of the optimal material AC-KOH and other reported materials are also summarized (Table 3).

**Table 1:** Element composition of AC-KOH and the control commercial YP50 by XPS analysis.

**Sample**	**C (at%)**	**N (at%)**	**O (at%)**
AC-KOH	94.46	0.67	4.87
YP50	94.06	0.51	5.43

**Table 2:** Detailed data for the calculation of E-SSA for AC-KOH electrode material.

***W***^***a***^**(nm)**	**DFT SSA (m**^**2**^**g**^**-1**^**)**	***D***^***b***^**(nm)**	**E-SSA (m**^**2**^**g**^**-1**^**)**
0.500	0	0.684	0
0.536	0	0.684	0
0.590	0	0.684	0
0.643	0	0.684	0
0.679	0	0.684	0
0.733	97	0.684	97
0.804	65	0.684	65
0.858	0	0.684	0
0.929	0	0.684	0
1.001	0	0.684	0
1.090	113	0.684	113
1.179	272	0.684	272
1.269	75	0.684	75
1.358	38	1.32	38
1.483	39	1.32	39
1.591	15	1.32	15
1.716	15	1.32	15
1.859	14	1.32	14
2.002	9	1.32	9
2.162	41	1.32	41
2.341	68	1.32	68
2.520	88	1.32	88
2.734	115	1.32	115
2.949	98	1.32	98
3.181	96	1.32	96
3.431	110	1.32	110
3.699	85	1.32	85
4.003	76	1.32	76
4.325	67	1.32	67
4.664	53	1.32	53
5.040	42	1.32	42
5.433	29	1.32	29
5.880	20	1.32	20
6.344	12	1.32	12
6.845	7	1.32	7
7.399	3	1.32	3
7.988	2	1.32	2
8.632	2	1.32	2
9.311	2	1.32	2
10.061	1	1.32	1
10.866	1	1.32	1
11.723	1	1.32	1
12.653	0	1.32	0
13.671	0	1.32	0
14.761	0	1.32	0
15.941	0	1.32	0
17.210	0	1.32	0
18.586	0	1.32	0
20.069	0	1.32	0
21.660	0	1.32	0
23.393	0	1.32	0
25.252	0	1.32	0
27.271	0	1.32	0
29.451	0	1.32	0
31.792	0	1.32	0
34.330	0	1.32	0
37.064	0	1.32	0
40.031	0	1.32	0
43.230	0	1.32	0
46.679	0	1.32	0
50.396	0	1.32	0
54.417	0	1.32	0
58.760	0	1.32	0
63.442	0	1.32	0
68.499	0	1.32	0
73.968	0	1.32	0
79.865	0	1.32	0
86.245	0	1.32	0
93.126	0	1.32	0
100.560	0	1.32	0
108.566	0	1.32	0
117.233	0	1.32	0
126.580	0	1.32	0
136.677	0	1.32	0
147.596	0	1.32	0

***W***^***a***^: pore width of the electrode material.

***D***^***b***^: diameter of the effective electrolyte ion.

**Table 3:** The structure and capacitive performance comparison between AC-KOH prepared in this work and other typical biomass derived ACs reported in literatures.

**Biomass**	**SSA (m**^**2**^**g**^**-1**^**)**	**Total pore volume (cm**^**3**^**g**^**-1**^**)**	***V***_**micro**_**(cm**^**3**^** g**^**-1**^**)**	***V***_**meso**_**(cm**^**3**^** g**^**-1**^**)**	***C***_**p**_**(F g**^**-1**^**)**	**Ref.**
egg white	3250	1.97	−	−	184 in LiPF_6_	[4]
ramie fiber	2087	1.08	0.43	0.65	287 in KOH	[5]
cotton fabric	777	0.2	−	−	184 in KOH	[6]
bamboo	1293	0.634	0.429	0.205	−	[7]
prosopis juliflora	2410	1.196	1.120	0.076	160 in LiPF_6_	[8]
sucrose	2953	1.26	1.03	0.23	160 in H_2_SO_4_	[9]
						
cornstalk core	2139	1.16	−	−	317 in KOH	[10]
					*E* = 6.8 W h kg^-1^	
					*P* = 28.3 kW kg^-1^	
sugar cane bagasse	2289	1.358	0.237	1.046	−	[11]
Starch	1157	0.97	0.05	0.92	144 in KOH	[12]
						
poplar catkins	1400	−	−	−	206 in aqueous electrolyte	[13]
					*E* = 7.5 W h kg^-1^	
						
pollen	3037	2.27	0.41	1.86	207 in AN	[14]
					*E* = 88 W h kg^-1^	
prosopis juliflora	2448	1.2116	1.1191	0.0925	−	[15]
corn grains	3199	1.645	1.015	0.63	257 in KOH	[16]
						
corncob	3054	1.50	0.738	0.762	328.4 in KOH	[17]
					401.6 in H_2_SO_4_	
acacia gum	1832	1.04	0.84	0.20	272 in KOH	[18]
corn stover	1671.4	0.831	0.634	0.197	236.4 in KOH	[19]
coconut kernel	1200	0.605	0.457	−	173 in H_2_SO_4_	[20]
potato starch	2342	1.24	1.08	0.16	335 in KOH	[21]
cow dung	1984	0.91	0.62	0.29	125 in AN	[22]
microalgae	2130	0.90	0.84	0.06	200 in LiCl	[23]
green needle coke	3347	1.8	−	−	348 in KOH	[24]
Sucrose	1941	0.919	0.874	0.045	148 in EMIMBF_4_	[25]
*β*-cyclodextrin	781	0.41	0.27	0.14	157 in KOH	[26]
dragon fruit skin	911	0.47	0.25	0.22	286.9 in KOH	[27]
						
corn straw	3237	2.27	0.42	1.85	222 in KOH	this work
					202 in TEABF_4_/AN	
					188 in EMIMBF_4_	
					*E* = 80 W h kg^-1^	
					*P* = 870 W kg^-1^	

## 2 Experimental design, materials and methods

AC-KOH and the control materials (AC-ZnCl_2_, AC-K_2_CO_3_, AC-Na_2_CO_3_, AC-KOH-2:1 and AC-KOH-5:1) were prepared through a hydrothermal carbonization and followed by an activation process using biomass straw as the raw material. A control carbon material, commercial activated carbon YP50, was obtained from Tianjin Plannano Energy Technologies Co., Ltd. The surface element composition of AC-KOH and YP50 was measured through XPS analysis using AXIS HIS 165 spectrometer (Kratos Analytical) with a monochromatized Al Kα X-ray source (1486.71 eV photons). As shown in Table 1, the contents of C and O elements in AC-KOH were estimated as 94.46% and 4.87% respectively, similar to that of commercial YP50. The PSD data of AC-KOH and the control carbon materials were obtained using the Brunauer-Emmett-Teller (BET) analysis and the density functional theory (DFT) method. Based on the PSD data, together with the cumulative DFT SSA and the electrolyte ion size, the E-SSA of electrode material was calculated. The electrochemical performance data, including cyclic voltammetry curves (CV), galvanostatic charge-discharge curves and cycling stability data, was obtained through assembled symmetrical coin-type supercapacitor.

Based on the PSD data of AC-KOH, together with the cumulative DFT SSA and the electrolyte ion size, the E-SSA of electrode material was calculated. The detailed data is shown in Table 2. The data of DFT SSA was obtained from the BET analysis directly. For TEABF_4_/AN electrolyte, the solvent free/bare TEA^+^ ion (diameter of 0.684 nm) and solvated TEA^+^ ion (diameter of 1.32 nm) were used for the E-SSA calculation [2], [3]. When the pore width of the carbon material is larger than 0.684 nm but smaller than 1.32 nm, the size of bare TEA^+^ ion was used for the calculation. While when the pore width of the carbon material is larger than 1.32 nm, the size of solvated TEA^+^ ion was used for such calculation. These two parts were then added together as the total accessible SSA for the electrolyte ions and are regarded as the E-SSA, which is 1771 m^2^ g^-1^ for AC-KOH electrode material as an example.

The capacitance performance of AC-KOH and YP50 was measured through two electrode system (coin-type supercapacitor). Fig. 2 shows the specific capacitance of the capacitors at different current density. In the TEABF_4_/AN electrolyte system, the specific capacitance of AC-KOH based capacitor is 206, 202 and 198 F g^-1^ at 0.5, 1 and 2 A g^-1^, respectively. Accordingly, YP50 based capacitor shows 101, 100 and 100 F g^-1^ at 0.5, 1 and 2 A g^-1^, respectively. The galvanostatic charge/discharge curves for AC-KOH based capacitor at current density of 5, 10, 15, 20 and 30 A g^-1^ are presented in Fig. 3.

The CV curves, galvanostatic charge/discharge curves, rate performances and Nyquist plots of AC-KOH electrode material in 6 M KOH electrolyte are shown in Fig. 4. The CVs (Fig. 4a) were measured in the voltage range of 0−1.0 V at the scan rates of 5, 10 and 20 mV s^-1^. The galvanostatic charge/discharge curves at different current densities are presented in Fig. 4b. The capacitance of AC-KOH of YP50 at current density of 0.1 A g^-1^ is 260 and 132 F g^-1^, respectively. The specific capacitance of AC-KOH is 216 F g^-1^ at current density of 2 A g^-1^ (Fig. 4c). The charge transfer resistance and ion diffusion performance were evaluated by the electrochemical impedance spectroscopy (EIS) measurements at a frequency range of 100 kHz to 10 mHz, as shown in Fig. 4d.

The galvanostatic charge/discharge curves for AC-KOH and YP50 based supercapacitors in EMIMBF_4_ electrolyte system tested at current density of 1 A g^-1^ are presented in Fig. 5. According to these curves, the capacitance of AC-KOH and YP50 based supercapacitors was calculated, which is 188 and 120 F g^-1^, respectively. The energy density of AC-KOH device, calculated using the formula *E*_cell_ = *C*_p_*V*^2^/8 (where *C*_p_ (F g^-1^) is the specific capacitance of the device and *V* (V) is the voltage), is 80 W h kg^-1^ at power density of 870 W kg^-1^, and the data of YP50 is 51 W h kg^-1^ at power density of 870 W kg^-1^. The power density, *P* (W kg^-1^), was calculated according to the formula *P* = *E*/Δ*t* (where *E* (W h kg^-1^) is the energy density of the device and Δ*t* (s) is the discharge time).

The application of an AC-KOH based coin-type supercapacitor cell for lighting a LED is shown in Fig. 6 and the supplementary video. The coin-type supercapacitor was assembled using AC-KOH as the electrode material and ionic liquid (EMIMBF_4_) as the electrolyte. The device firstly was charged at 1 A g^-1^ to 3.5 V and then used to light the LED with a working potential around 2.2 V and working power about 40 mW. The LED could maintain light for ~ 30 minutes.

In addition, the structure and capacitive performance data of AC-KOH material in this work and the comparison data with the reported reports are summarized in Table 3, as shown in below.

## References

[1] Lu Y., Zhang S., Yin J., Bai C., Zhang J., Li Y., Yang Y., Ge Z., Zhang M., Wei L., Ma M., Ma Y., Chen Y. (2017). Mesoporous activated carbon materials with ultrahigh mesopore volume and effective specific surface area for high performance supercapacitors. Carbon.

[2] Zhang L., Yang X., Zhang F., Long G., Zhang T., Leng K., Zhang Y., Huang Y., Ma Y., Zhang M., Chen Y. (2013). Controlling the effective surface area and pore size distribution of sp^2^ carbon materials and their impact on the capacitance performance of these materials. J. Am. Chem. Soc..

[3] Lu Y., Long G., Zhang L., Zhang T., Zhang M., Zhang F., Yang Y., Ma Y., Chen Y. (2016). What are the practical limits for the specific surface area and capacitance of bulk sp^2^ carbon materials. Sci. China Chem..

[4] Li B., Dai F., Xiao Q., Yang L., Shen J., Zhang C., Cai M. (2016). Activated carbon from biomass transfer for high-energy density lithium-ion supercapacitors. Adv. Energy Mater..

[5] Du X., Zhao W., Wang Y., Wang C., Chen M., Qi T., Hua C., Ma M. (2013). Preparation of activated carbon hollow fibers from ramie at low temperature for electric double-layer capacitor applications. Bioresour. Technol..

[6] Chen L., Ji T., Mu L., Zhu J. (2017). Cotton fabric derived hierarchically porous carbon and nitrogen doping for sustainable capacitor electrode. Carbon.

[7] Kim Y.J., Lee B.J., Suezaki H., Chino T., Abe Y., Yanagiura T., Park K.C., Endo M. (2006). Preparation and characterization of bamboo-based activated carbons as electrode materials for electric double layer capacitors. Carbon.

[8] Sennu P., Choi H.-J., Baek S.-G., Aravindan V., Lee Y.-S. (2016). Tube-like carbon for Li-ion capacitors derived from the environmentally undesirable plant: prosopis juliflora. Carbon.

[9] Wei L., Yushin G. (2011). Electrical double layer capacitors with activated sucrose-derived carbon electrodes. Carbon.

[10] Liu C., Han G., Chang Y., Xiao Y., Li M., Zhou W., Fu D., Hou W. (2016). Properties of porous carbon derived from cornstalk core in high-performance electrochemical capacitors. Chemelectrochem.

[11] Liou T.-H. (2010). Development of mesoporous structure and high adsorption capacity of biomass-based activated carbon by phosphoric acid and zinc chloride activation. Chem. Eng. J..

[12] Wu M., Ai P., Tan M., Jiang B., Li Y., Zheng J., Wu W., Li Z., Zhang Q., He X. (2014). Synthesis of starch-derived mesoporous carbon for electric double layer capacitor. Chem. Eng. J..

[13] Wei Y. (2014). Activated carbon microtubes prepared from plant biomass (poplar catkins) and their application for supercapacitors. Chem. Lett..

[14] Zhang L., Zhang F., Yang X., Leng K., Huang Y., Chen Y. (2013). High-performance supercapacitor electrode materials prepared from various pollens. Small.

[15] Sennu P., Aravindan V., Ganesan M., Lee Y.-G., Lee Y.-S. (2016). Biomass-derived electrode for next generation lithium-ion capacitors. Chemsuschem.

[16] Balathanigaimani M.S., Shim W.-G., Lee M.-J., Kim C., Lee J.-W., Moon H. (2008). Highly porous electrodes from novel corn grains-based activated carbons for electrical double layer capacitors. Electrochem. Commun..

[17] Wang D., Geng Z., Li B., Zhang C. (2015). High performance electrode materials for electric double-layer capacitors based on biomass-derived activated carbons. Electrochim. Acta.

[18] Fan Y., Liu P., Zhu B., Chen S., Yao K., Han R. (2015). Microporous carbon derived from acacia gum with tuned porosity for high-performance electrochemical capacitors. Int. J. Hydrog. Energy.

[19] Jin H., Wang X., Shen Y., Gu Z. (2014). A high-performance carbon derived from corn stover via microwave and slow pyrolysis for supercapacitors. J. Anal. Appl. Pyrolysis.

[20] Kishore B., Shanmughasundaram D., Penki T.R., Munichandraiah N. (2014). Coconut kernel-derived activated carbon as electrode material for electrical double-layer capacitors. J. Appl. Electrochem.

[21] Zhao S., Wang C.-Y., Chen M.-M., Wang J., Shi Z.-Q. (2009). Potato starch-based activated carbon spheres as electrode material for electrochemical capacitor. J. Phys. Chem. Solids.

[22] Bhattacharjya D., Yu J.-S. (2014). Activated carbon made from cow dung as electrode material for electrochemical double layer capacitor. J. Power Sources.

[23] Sevilla M., Gu W., Falco C., Titirici M.M., Fuertes A.B., Yushin G. (2014). Hydrothermal synthesis of microalgae-derived microporous carbons for electrochemical capacitors. J. Power Sources.

[24] Wang J., Chen M., Wang C., Wang J., Zheng J. (2011). Preparation of mesoporous carbons from amphiphilic carbonaceous material for high-performance electric double-layer capacitors. J. Power Sources.

[25] Wei L., Yushin G. (2011). Electrical double layer capacitors with sucrose derived carbon electrodes in ionic liquid electrolytes. J. Power Sources.

[26] Feng S., Li W., Wang J., Song Y., Elzatahry A.A., Xia Y., Zhao D. (2014). Hydrothermal synthesis of ordered mesoporous carbons from a biomass-derived precursor for electrochemical capacitors. Nanoscale.

[27] Feng W., He P., Ding S., Zhang G., He M., Dong F., Wen J., Du L., Liu M. (2016). Oxygen-doped activated carbons derived from three kinds of biomass: preparation, characterization and performance as electrode materials for supercapacitors. RSC Adv..

